# Characterization of the Tellurite-Resistance Properties and Identification of the Core Function Genes for Tellurite Resistance in *Pseudomonas citronellolis* SJTE-3

**DOI:** 10.3390/microorganisms10010095

**Published:** 2022-01-01

**Authors:** Wanli Peng, Yanqiu Wang, Yali Fu, Zixin Deng, Shuangjun Lin, Rubing Liang

**Affiliations:** 1 State Key Laboratory of Microbial Metabolism, School of Life Sciences and Biotechnology, Shanghai Jiao Tong University, Shanghai 200240, China; chinapengwl@sjtu.edu.cn (W.P.); wyq501194864@163.com (Y.W.); fuyali_1502@163.com (Y.F.); zxdeng@sjtu.edu.cn (Z.D.); linsj@sjtu.edu.cn (S.L.); 2 Joint International Research Laboratory of Metabolic and Developmental Sciences, School of Life Sciences and Biotechnology, Shanghai Jiao Tong University, Shanghai 200240, China

**Keywords:** tellurite resistance, *terZABCDE* gene cluster, core function gene, *terC* gene, *terD* gene, *terA* gene

## Abstract

Tellurite is highly toxic to bacteria and commonly used in the clinical screening for pathogens; it is speculated that there is a potential relationship between tellurite resistance and bacterial pathogenicity. Until now, the core function genes of tellurite resistance and their characteristics are still obscure. *Pseudomonas citronellolis* SJTE-3 was found able to resist high concentrations of tellurite (250 μg/mL) and formed vacuole-like tellurium nanostructures. The *terZABCDE* gene cluster located in the large plasmid pRBL16 endowed strain SJTE-3 with the tellurite resistance of high levels. Although the *terC* and *terD* genes were identified as the core function genes for tellurite reduction and resistance, the inhibition of cell growth was observed when they were used solely. Interestingly, co-expression of the *terA* gene or *terZ* gene could relieve the burden caused by the expression of the *terCD* genes and recover normal cell growth. TerC and TerD proteins commonly shared the conserved sequences and are widely distributed in many pathogenic bacteria, highly associated with the pathogenicity factors.

## 1. Introduction

Tellurium (Te) is a rare metalloid that belongs to the chalcogen family, whose abundance is relatively low in the earth’s crust (about 0.027 ppm) [1]. As a p-type semiconductor, Te is commonly used in the manufacture of solar panels, glass, rubber, photocopiers, and metal alloys [2,3,4]. It was considered one of the two most important materials according to the Materials Genome Initiative launched by the United States of America.

In nature, tellurium is mostly in the form of alloys with other metals like copper. Tellurite (TeO_3_^2^^−^) and tellurate (TeO_4_^2^^−^) are the most common forms of tellurium in ecological environments [5,6]. Tellurite is highly toxic to various creatures, especially bacteria. Very low concentration (1 µg/mL) of tellurite can severely inhibit cell growth of *E. coli*, much lower than that of heavy metal ions [7]. Therefore, tellurite has been used as an antibacterial agent before the use of penicillin. Several hypotheses have been presented to explain the toxicity mechanism of tellurite to bacteria, and therein ROS stress is supposed to play a major role in tellurite toxicity [8,9,10,11,12].

Meanwhile, some microorganisms have been found with the tellurite-resistance capability. Several pathogenic bacteria like *E. coli* O157:H7, *Diphtheria bacillus*, *Staphylococcus aureus*, *Shigella* spp., and *Streptococcus pneumoniae* showed different resistance to tellurite [7]. In fact, tellurite has been effectively and widely used for the screening of highly pathogenic microbes in clinical tests for over 50 years [7]. When streaked on the tellurite-amended plates, the pathogenic strains can reduce tellurite into the elemental Te particles and appear as black or grey colonies. It implied that these pathogenic strains may contain specific genes functioning in the transportation and reduction of tellurite.

The potential genes responsible for tellurite resistance were first identified in bacteria, and their homologs were also found in over 50 different species including plants and animals. The reported tellurite-resistance genes and gene clusters include *pacB*, *tmp*, *tehAB*, *kilAtelAB*, *arsABC*, *narGHI*, *trgABcysK*, and *terZABCDE*; the combination of potential tellurite-resistance genes varied greatly in different strains [11,13,14,15,16,17,18]. The *terZABCDE* gene cluster is considered as an intact gene cluster for tellurite resistance, ensuring the highest tellurite resistance with the minimal inhibition concentration (MIC) of 1024 μg/mL, which has been identified in various pathogens like the enteropathogenic *E. coli*, *P. aeruginosa,* and *Y. pestis* [18].

Several attempts have been performed to study the functions of tellurite-resistance genes and clarify their roles. The membrane protein TerC was classified into LysE superfamily, which is related to the export of manganese; a riboswitch responding to manganese ions was also found upstream of the *terC* gene [19,20]. TerC was speculated to participate in the transportation of tellurite within and outside cells [21]. The TerB protein from *K. pneumoniae* was the first tellurite-resistance protein with the resolved structure, containing the N-terminal positively charged domain and the C-terminal negatively charged domain [22]. The TerD protein from *K. pneumoniae* able to bind Ca^2+^ and the Ca^2+^ signaling was supposed to play a role in the bacterial tellurite resistance [23]. Besides, several genes involved in cysteine metabolism are also considered to be probably associated with tellurite resistance [12]. However, although several tellurite resistance genes have been analyzed, the role of each gene and the core function genes is still obscure.

*Pseudomonas. citronellosis* is one species isolated from the natural environment and the reported strains are the nonpathogenic gram-negative bacteria [24]. *P. citronellosis* SJTE-3 is found with efficient pollutant-degrading capacities and contains a *terZABCDE* gene cluster in its plasmid pRBL16 [25,26]. In this work, the tellurite-resistance properties of *P. citronellolis* SJTE-3 were studied, and the core function genes for tellurite resistance were identified. The conservation and distribution of core tellurite-resistance genes were further analyzed; the conceivable relevance of tellurite resistance and bacterial pathogenicity was also discussed. The findings can facilitate the elucidation of bacterial tellurite-resistance mechanism and the clarification of its function in pathogens.

## 2. Materials and Methods

### 2.1. Strains, Plasmids, Media, and Chemicals

All strains and plasmids used in this study are listed in Table S1. LB medium (tryptone 10.0 g/L, yeast extract 5.0 g/L, NaCl 8.0 g/L) was used for strain culture and solid plates were prepared by supplying agar (15.0 g/L) to the liquid medium. The minimal medium (KH_2_PO_4_ 4.5 g/L, K_2_HPO_4_·3H_2_O 13.75 g/L, (NH_4_)_2_SO_4_ 2.0 g/L, MgSO_4_·7H_2_O 0.16 g/L, FeSO_4_ 5.0 µg/L, CaCl_2_·2H_2_O 1.0 µg/L, MnCl_2_·4H_2_O 2.0 µg/L, pH 7.4) was used for plasmid curing. Potassium tellurite (K_2_TeO_3_) was dissolved in Millipore water to the concentration of 10 mg/mL and stored at −20 °C. All chemicals were of analytical grade, purchased from Sangon Biotech Ltd. (Shanghai, China).

### 2.2. Standard DNA Operation

The oligonucleotides used in this work were synthesized by Sangon Biotech Ltd. (Shanghai, China) and listed in Table S2. PCR amplification was performed following the standard protocols. DNA polymerases were purchased from TaKaRa Co. (Dalian, China); T4 DNA ligases and the restriction endonucleases were purchased from Thermo Fisher Scientific Inc. (Waltham, MA, USA). The *Xba* I/*Bam* HI-treated PCR fragments and vector frameworks were ligated with T4 DNA ligase and transformed into *E. coli* DH5α competent cells. The transformants were screened on the LB plates with gentamicin and the positive recombinant plasmids were verified by DNA sequencing. The sequencing of DNA fragments and plasmid DNAs were performed with an ABI 3730 xl DNA Analyzer sequenator from Thermo Fisher Scientific Inc. (Waltham, MA, USA) using the standard DNA analysis buffer system at Sangon Biotech Ltd. (Shanghai, China); the fragment sizes were then analyzed using the Peakscanner software (Thermo Fisher Scientific). The chemical transformation and the electroporation-mediated transformation were used to transform plasmids into *E. coli* strains and *Pseudomonas* strains. The genome DNA, plasmid DNA, and PCR fragments were obtained with the TIANamp Bacteria DNA Kit, TIANprep Mini Plasmid Kit, and TIANquick Mini Purification Kit, respectively (Tiangen, Beijing, China).

### 2.3. Bioinformatics Analysis

The sequences of the tellurite-resistance genes or gene clusters in different bacteria were obtained from National Center for Biotechnology Information (NCBI), and their evolutionary distances were analyzed by MEGA X [27]. The multiple sequence alignments (MSA) of the tellurite resistance genes and proteins were performed with Mauve and DNAMAN software with default parameters.

### 2.4. Combination of the Tellurite-Resistance Genes

To determine the core function genes for tellurite resistance in *P. citronellolis* SJTE-3, the fragments of potential tellurite-resistance genes were amplified from plasmid pRBL16 with the corresponding primers (Table S2). The upstream 500 bp region of the *terZABCDE* gene cluster was cloned into pBSPPc as a wild-type promoter and generated plasmid pBS-Pter. Different gene combinations of the *terZABCDE* gene cluster were inserted into plasmid pBS-Pter under this promoter, generating a series of recombinant plasmids (Table S1). All plasmids were confirmed by sequencing with an ABI 3730 xl DNA Analyzer sequenator from Thermo Fisher Scientific Inc. (Waltham, MA, USA) using the standard DNA analysis buffer system at Sangon Biotech Ltd. (Shanghai, China).

### 2.5. Evaluation of Tellurite Resistance Properties

The cell growth and the tellurite-resistance properties of different strains in this work were determined using the agar and broth dilution methods as described before [28,29,30]. The cell growth curve and the MIC value of tellurite to cells were assessed after a defined period of incubation [30]. For *P. citronellolis* SJTE-3 and SJTE-3ΔpRBL16, the single colony was inoculated into 3 mL LB medium and cultured overnight, then inoculated into 100 mL LB medium. The cells were harvested at exponential phase and washed with Millipore water thrice; then the cell inocula were added into fresh LB medium amended with tellurite of different concentrations (0–500 μg/mL) and the initial OD_600_ is 0.05. The cells were cultured at 37 °C and sampled every 2 h; the samples were diluted and spread on a plate for overnight culture to calculate the colony-forming units (CFU). Three independent experiments were repeated, and the presented data were the average values.

The cell growth and tellurite resistance properties of *E. coli* MG1655 cells containing different plasmids were determined similarly as above. Cells at the exponential phase were collected and inoculated into fresh LB medium containing tellurite (0–100 μg/mL) with the initial OD_600_ of about 0.05. The growth curves were monitored with an Automated Microbiology Growth Curve Analyzer (Bioscreen, Oy Growth Curves Ab Ltd., Finland) at 37 °C for 24 h. The recombinant *E. coli* cells were also screened on the tellurite-amended plates. Strains able to grow on the solid plates with tellurite of certain concentrations and simultaneously grow in the liquid medium containing tellurite with OD_600_ value over 0.2 after 24 h, were considered capable of resisting tellurite of this specific concentration. Three independent experiments were performed and the average values were presented.

### 2.6. Transmission Electron Microscope (TEM) Observation

A single colony of *P. citronellolis* SJTE-3 or *E. coli* DH5α was inoculated into LB medium and cultured overnight. After being collected and washed with the sterile water, the cell inocula were cultured in the minimal medium to the exponential phase. Then 10 μg/mL K_2_TeO_3_ was added and the culture was incubated for 2 h. The collected cells were then washed with PBS buffer thrice. After the cells were gradually dehydrated using the resin of different concentrations (30–100%), they were embedded and sectioned in a cryo-ultramicrotome (Leica Instruments GmbH, Wetzlar, Germany). The embedded cell samples were examined under a 120-kV Biology Transmission Electron Microscope (Thermo Fisher Scientific Inc., MA, USA), and the cell morphologies were captured.

## 3. Results

### 3.1. Plasmid pRBL16 Endowed P. citronellolis SJTE-3 with High-Level Tellurite Resistance

*P. citronellolis* SJTE-3 was an environment-isolated pollutant degrading strain with clear genetic background (GenBank No. NZ_CP015879) [25,26]. Genome analysis showed that there is a *terZABCDE* gene cluster in its plasmid pRBL16, which might confer this strain with tellurite resistance.

**Figure 1:**
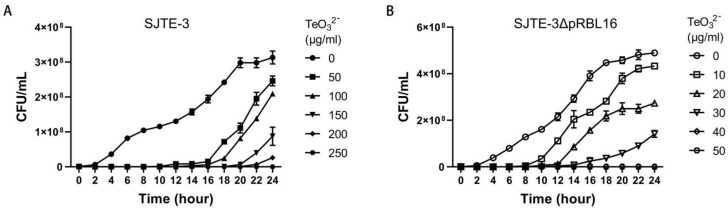
The cell growth curves of *P. citronellolis* SJTE-3 and SJTE-3ΔpRBL16 cultured with tellurite of different concentrations. Strain SJTE-3 (**A**) and strain SJTE-3ΔpRBL16 (**B**) were cultured in liquid media with tellurite (from 0 to 500 μg/mL) and solid plates with tellurite (from 0 to 500 μg/mL). The cell growth was detected every 2 h and showed in CFU/mL. Three independent experiments were performed and the average values were calculated with standard bars.

**Figure 2:**
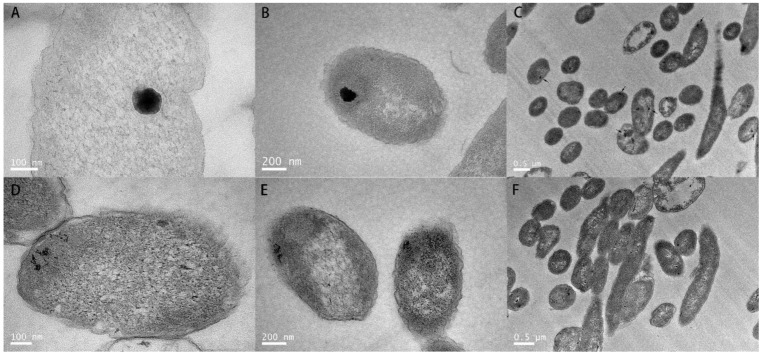
The TEM images of *P. citronellolis* SJTE-3 and *E. coli* DH5α cells cultured with tellurite. Strain SJTE-3 (**A**–**C**) and DH5α (**D**–**F**) were incubated in liquid medium and cultured to an exponential phase. After being supplied with 10 μg/mL tellurite and cultured for 2 h, the cells were collected, treated, and observed with TEM. The images of the cells were photographed at different magnifications.

To determine the role of plasmid pRBL16 in the tellurite resistance of strain SJTE-3, the plasmid cured strain SJTE-3ΔpRBL16 was generated and their tellurite resistance properties were analyzed. Although the two strains grew normally and both formed black colonies on tellurite amended plates, the wild-type strain SJTE-3 showed much higher tellurite resistance than strain SJTE-3ΔpRBL16 (Figure 1). The MIC value of tellurite to strain SJTE-3 was about 250 μg/mL, much higher than those of normal lab-used *E. coli* strains lacking the tellurite resistance genes and plasmids (MIC about 0.5 μg/mL of strains DH5α and MG1655) and those of most strains reported with tellurite resistance (Table S3). After removing plasmid pRBL16, the MIC of tellurite to strain SJTE-3ΔpRBL16 decreased to only about 40 μg/mL (Figure 1). It demonstrated that plasmid pRBL16 is the primary cause of high-level tellurite resistance for strain SJTE-3. Further TEM detection showed that strain SJTE-3 reduced tellurite into smooth and even vascular-like tellurium nanoparticles with about 100 nanometers in diameter; while the particles of tellurium formed by *E. coli* DH5α cells were small and loose (Figure 2). This meant that there exist specific genes for transmembrane transportation and efficient reduction of tellurite in *P. citronellolis* SJTE-3.

### 3.2. The terZABCDE Gene Clusters in Different Bacteria May Have the Same Origin

The properties of plasmid pRBL16 were analyzed to understand the genetic basis of tellurite resistance in *P. citronellolis* SJTE-3. Plasmid pRBL16 contains 512 ORFs with 56.67% GC content (Table 1). It shares a common backbone of the megaplasmid family carrying large arrays of antibiotic resistance genes in *Pseudomonas*, while it lacks the antimicrobial resistance (AMR) genes [31]. Due to the difference in the average GC content between plasmid (56.72%) and chromosome (67.99%), it was thought that plasmid pRBL16 may come from horizontal transfer. The top functional COG categories of plasmid genes were signal transduction mechanisms, replication, ribosomal structure and biogenesis, intracellular trafficking, secretion, and vesicular transport. The plasmid genes may function as virulence factors (92 genes), antibiotic resistance (64 genes), heavy metal resistance (20 genes), ROS resistance (23 genes), and secretion (34 genes). Except for the *terZABCDE* gene cluster, there are still other resistance genes like copper resistance genes, mercury resistance genes, and silver resistance genes in plasmid pRBL16, which may endow strain SJTE-3 with the resistance to different metals. Part of *arsABC* genes, *narGHI* genes, and *cysK* gene are also aligned in the genome, probably contributing to the tellurite resistance of strain SJTE-3ΔpRBL16 [32,33,34].

**Figure 3:**
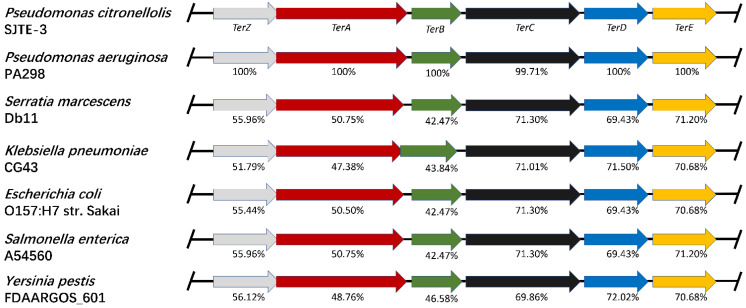
Comparison of the tellurite-resistance gene clusters in different bacteria. The *terZABCED* gene clusters in the plasmids from *P. citronellolis* SJTE-3, *P. aeruginosa* PA298, *Serratia marcescens* CG43, *E. coli* O157, *S. enterica* A54560, and *Y. pestis* FDAARGOS_601 were aligned with Mauve. The *terZABCDE* genes were labeled in gray, red, green, black, blue, and yellow, respectively. The percentage represented the similarity of tellurite-resistance proteins from other bacteria to those from strain SJTE-3.

As plasmid pRBL16 in strain SJTE-3 is probably obtained by horizontal transfer, it was thought whether the tellurite-resistance genes or gene clusters in different bacteria come from a close origin and share similar sequences. Therefore, the homologous gene clusters for tellurite resistance from different species were aligned and analyzed with Mauve. Results showed that the *terZABCDE* gene clusters in different strains share relatively conserved sequences (Figure 3). The *terCDE* genes from different species shared about 70% similarities, while the *terZAB* genes shared more than 42% similarities (Figure 3). Besides, the TerD and TerE proteins from *P. citronellolis* SJTE-3 share very high sequence identity (66.67%) with each other and may execute similar functions. These results indicate that the *terZABCDE* gene clusters in different species are likely to evolve from close origination and share conserved sequences. As the conservation of *terC*, *terD* and *terE* genes are much higher than those of *terZ*, *terA,* and *terB* genes, the first three genes may be vital for tellurite resistance.

### 3.3. The terCD Genes Are the Minimal Function Combination for Tellurite Resistance

**Figure 4:**
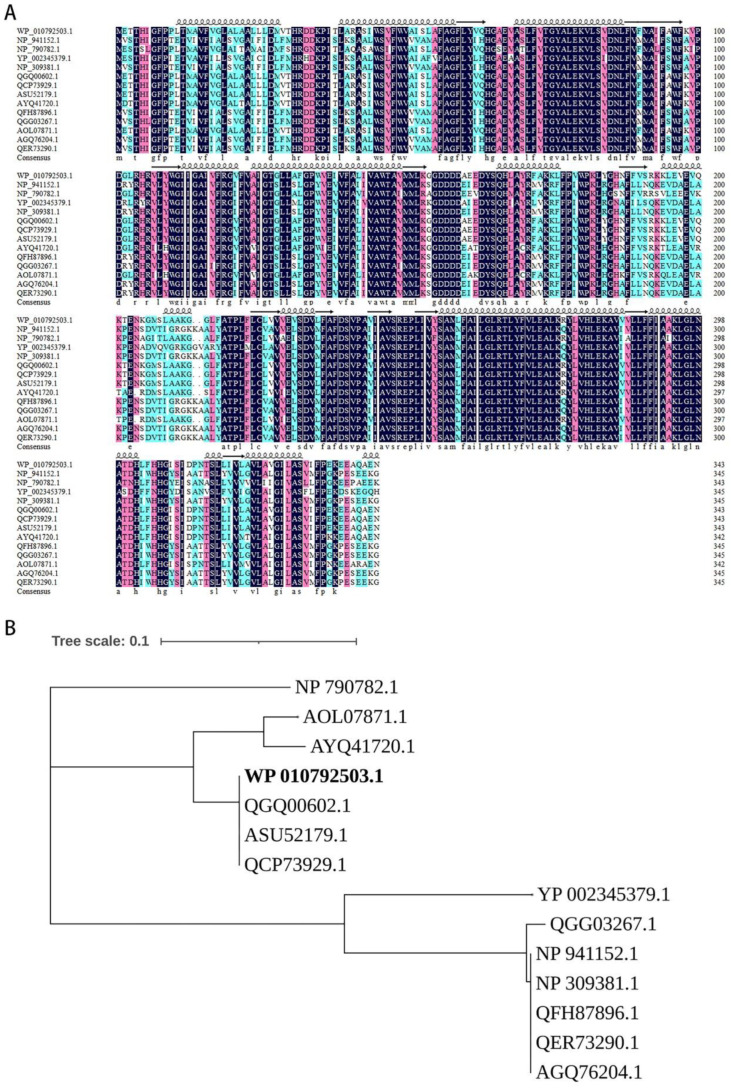
The multiple sequences alignment and the phylogenetic analysis of TerC. (**A**) The sequences of TerC were from *P. citronellolis* SJTE-3 (WP_010792503.1, bold marked), *Serratia marcescens* R478 (NP_941152.1), *P. syringae* DC3000 (NP_790782.1), *Y. pestis* CO92 (YP_002345379.1), *E. coli* O157:H7 str. Sakai (NP_309381.1), *P. aeruginosa* T2436 (QGQ00602.1), *P. aeruginosa* A298 (QCP73929.1), *P. putida* SY153 (ASU52179.1), *Burkholderia lata* A05 (AYQ41720.1), *Enterobacter hormaechei* E5 (QFH87896.1), *Cronobacter sakazakii* CFSAN068773 (QGG03267.1), *Burkholderia contaminans* FL-1-2-30-S1-D0 (AOL07871.1), *S. enterica* CFSAN002050 (AGQ76204.1), *E. coli* ST95-32 (QER73290.1). The conserved amino acids were marked black and the secondary motifs were marked. (**B**) The phylogenetic tree of TerC proteins was constructed in MEGA X using the Neighbor-Joining method, and the bootstrap consensus tree was performed with 1000 replications.

**Figure 5:**
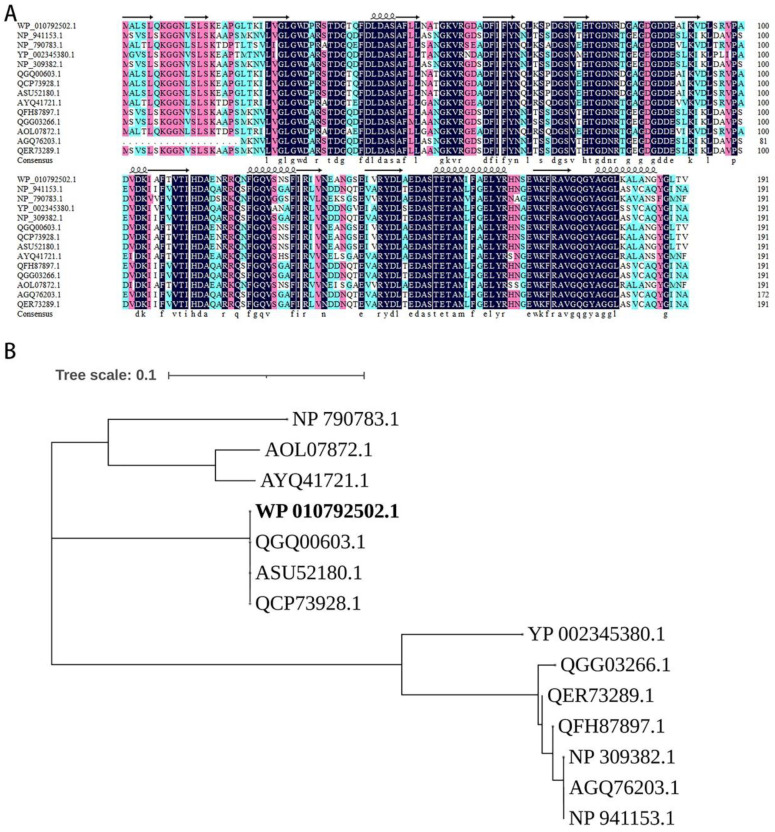
The multiple sequences alignment and the phylogenetic analysis of TerD. (**A**) The sequences of TerD were from *P. citronellolis* SJTE-3 (WP_010792502.1, bold marked), *Serratia marcescens* R478 (NP_941153.1), *P. syringae* DC3000 (NP_790782.1), *Y. pestis* CO92 (YP_002345380.1), *E. coli* O157:H7 str. Sakai (NP_309381.1), *P.aeruginosa* T2436 (QGQ00603.1), *P.* *aeruginosa* A298 (QCP73928.1), *P. putida* SY153 (ASU52180.1), *Burkholderia lata* A05 (AYQ41721.1), *Enterobacter hormaechei* E5 (QFH87897.1), *Cronobacter sakazakii* CFSAN068773 (QGG03266.1), *Burkholderia contaminans* FL-1-2-30-S1-D0 (AOL07872.1), *S. enterica* CFSAN002050 (AGQ76203.1), *E. coli* ST95-32 (QER73289.1). The conserved amino acids were marked black and the secondary motifs were marked. (**B**) The phylogenetic tree of these TerD proteins was constructed in MEGA X using the Neighbor-Joining method, and the bootstrap consensus tree was performed with 1000 replications.

Although some tellurite-resistance genes have been identified, their components and combination varied in various species and the core function genes are still unclear. To identify the core genes for tellurite resistance, genes in the *terZABCDE* gene cluster in plasmid pRBL16 were combined randomly (from 2 genes to 5 genes), cloned into plasmid pBS-Pter under the promoter of *ter* gene cluster, and then transformed into *E. coli* DH5α cells to generate a series of transformants. The resistance of these transformants to tellurite of different concentrations (1–100 μg/mL) was presented with cell growth state, listed in Table 2. Results indicated that all recombinant strains containing *terCD* (or *terCE*) genes could resist tellurite of high concentration, while the transformants containing other gene combinations without *terCD* or *terCE* genes did not show tellurite resistance (Table 2). This indicated that *terC* and *terD* (*terE*) genes were the core function genes for tellurite resistance in strain SJTE-3. However, the transformants containing plasmids pBS-*terCD* or pBS-*terCE* could only form very tiny colonies, even cultured on LB plate without tellurite for 24 h. Interestingly, this growth retardation could be efficiently rescued by extra expression of *terA* gene or *terZ* gene in the cluster. The transformants containing plasmids with *terACD* or *terZCD* genes showed high levels of tellurite resistance and robust cell growth (Table 2). Further MSA analysis indicated that TerC and TerD proteins from different species shared high sequence similarities (84.29% and 83.63%). The evolutionary analyses also showed that the homologs of the two proteins in different species had a close evolutionary distance (Figure 4 and Figure 5). Therefore, despite the cell growth burden, *terCD* genes were supposed to be the core function genes for tellurite resistance; *terA* and *terZ* genes also played an unknown but important role in the bacterial resistance to tellurite.

### 3.4. The terCD Genes and Pathogenic Genes Are in Co-Distribution

**Figure 6:**
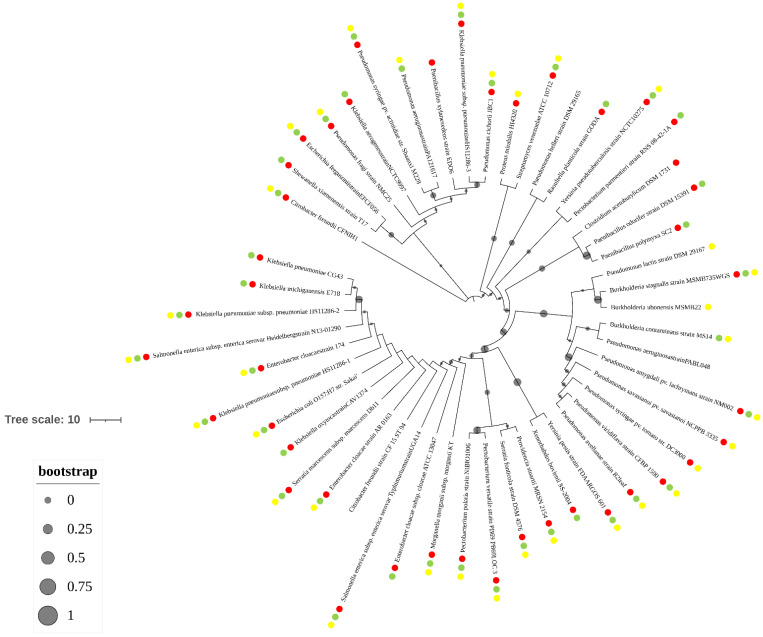
The phylogenetic and the co-existence analysis of *terZABCDE* gene cluster and the pathogen-related genes. The phylogenetic tree of *terZABCDE* gene cluster in different pathogenic bacteria was constructed in MEGA using the Neighbor-Joining method, and the bootstrap consensus tree was performed with 1000 replications. The existence of genes encoding siderophore, the virulence protein, and the type III secretion system are shown by the red points, green points, and yellow points.

As tellurite is commonly used in the clinical screening of highly pathogenic bacteria, it was speculated that there may exist a correlation between tellurite resistance and microbial pathogenicity. However, analysis on the distribution of tellurite-resistance genes and pathogenic related genes was rarely reported. Therefore, the distribution of the core tellurite-resistance genes (*terCD*) and the pathogenic related genes in different pathogenic bacteria were analyzed, and their evolutionary distance was also calculated (Figure 6). Results indicated that most of the pathogenic strains containing *terCD* genes often simultaneously contained the genes encoding various pathogenic factors like virulence proteins, siderophores, and type III secretion systems (Figure 6). The co-distribution of *terCD* genes and pathogenic factors genes are commonly existed and highly associated, supporting the relevance speculation of tellurite resistance and microbial pathogenicity.

## 4. Discussion

Due to its high toxicity to bacteria, tellurite has been used as an antibacterial agent and in clinical screening for highly pathogenic strains [7]. The *terZABCDE* gene cluster for tellurite resistance has been found located in plasmid DNA or chromosome DNA of many microorganisms, sharing conserved sequences and close origin [18,35,36,37]. Although the tellurite-resistance genes are supposedly highly relevant to pathogenicity, the core function genes for tellurite resistance and its genetic relevance between tellurite resistance and bacterial pathogenicity are indistinct [30,36,38,39,40,41,42].

The tellurite-resistance gene cluster (*terZABCDE*) in *P.* *citronellolis* SJTE-3 was found in its plasmid pRBL16, which was classified as pBT2436-like megaplasmid commonly existed in pathogens; and the tellurite-resistance genes were identified as part of the core genes in this megaplasmid family. However, plasmid pRBL16 was isolated from non-human sources and lack of any antimicrobial resistance genes [31]. Therefore, the pathogenic origination of plasmid pRBL16 and nonpathogenic properties of *P.* *citronellolis* make strain SJTE-3 as an ideal candidate for the study of core function genes for tellurite resistance.

The *terCD* genes were identified as the core function gene for the high-level resistance to tellurite in *P. citronellolis* SJTE-3, although their overexpression caused obvious retardation in cell growth. Recombinant plasmids carrying *terCE* genes endowed cells with high tellurite resistance, similar to plasmids containing *terCD* genes did (Table 2). As TerD and TerE are homologous proteins, it is supposed that they derived from gene duplication and executed similar functions. On the other hand, the co-existence of TerD and TerE implied that a single *terD* gene may not be sufficient to achieve efficient tellurite resistance. It was also noticed that the recombinant strains containing *terE* gene usually showed weaker tellurite resistance and formed smaller colonies than those with *terD* gene, implying the similar but not identical function of *terD* and *terE* genes.

The growth burden observed in strains containing *terCD* genes may be due to the lack of regulatory factors or unknown detoxic factors for the integrity of the gene cluster [43]. This cell growth burden could be rescued when the *terA* gene or *terZ* gene was co-expressed with *terCD* genes. All the transformants containing *terACD* or *terZCD* in plasmid showed vigorous cell growth and high tellurite resistance. Therefore, *terA* and *terZ* genes were supposedly important for tellurite resistance as well. The TerA and TerZ proteins are classified as TerD family proteins according to their conserved TerD-like domains. TerA protein contains a TerD-like super-family domain, a TerD-like domain, and two putative metal-binding sites; TerZ protein contains a TerD-like domain and a putative metal-binding site. It implied that the TerD-like domain and metal ions may be important for the improvement of bacterial cell state to resist tellurite. TerB protein previously was considered as a central unit of the *terZABCDE* gene cluster [44]. However, our results indicated that *terB* gene in *P. citronellolis* SJTE-3 was not necessary for the acquisition of tellurite resistance of *E. coli* MG1655 (Table 2).

TerC protein is a hydrophobic protein and was speculated as a membrane protein probably involved in the transportation of tellurite [21]. It is also the only membrane protein in *terZABCDE* gene cluster, implying its function may be irreplaceable. Proteomics analysis of TerC interactome suggests that the TerC-TerB complex may act as the central unit, and TerD may participate in tellurite resistance via a TerC-independent action [44]. Structure analysis of TerD protein from *K. pneumoniae* showed that there are two Ca^2+^ binding sites in this protein, the Ca1 site (formed by residues 40, 41, 42, 78, 79, and 89) and the Ca2 site (formed by residues 29, 82, 84, and 88) [23]. MSA analysis in this work showed that the predicted metal-binding sites of TerD are conserved through different species. It suggested that the binding of Ca^2+^ was probably vital for the function of TerD protein, which may be regulated by the calcium signal transduction [23].

In fact, *terC* and *terD* genes in different species are identified with various functions. The TerC homologue identified in the thylakoid membrane of *Arabidopsis thaliana* was proposed to play a key role in the formation of chloroplast [45,46]. The *terC* genes from various bacteria were located in the downstream of *yybP-ykoY* magnesium ions responsive riboswitch, and TerC protein also shows high similarity to manganese transporter MntP and MneA, suggesting that TerC may be involved in the metal transportation in bacteria [19,20]. Although it is hard to precisely predict the role of TerC in tellurite resistance, it seems that TerC protein was vital for the transportation of tellurite and other metal ions in bacteria. The TerD protein has been found to participate in the differentiation, cell growth, and spores forming of *S. coelicolor* [47]. It also is supported by the growth improvement of TerD-like proteins (TerA and TerZ) to the TerCD-containing cells in this work. Therefore, TerC and TerD proteins have versatile functions and tellurite resistance may be one of their functions [18,41,42].

In bacteria, *terZABCDE* genes also are involved in the resistance to bacteriophages, the phagocytosis by macrophages, the tolerance to oxidative stresses and colicins, the adherence to epithelial cells and the formation of filamentous morphology [33,35,38,48,49,50,51]. When the *terZABCDE* gene cluster from *Clostridium acetobutylicum* was expressed in *E. coli recA* mutant, cells even showed resistance to mitomycin C and methyl methanesulfonate [52]. Meanwhile, the *terD* and *terE* genes of *Y. pestis* were up-regulated when the bacteria proliferated in mouse macrophage phagolysosomes [53]. The transcription levels of *terA* and *terC* genes in *E. coli* EDL933 cells in human macrophages were also elevated [54]. These suggested that there may exist a potential relationship in tellurite resistance and cell survival during the bacterial infection process. The plasmid pRBL16 in strain SJTE-3 is a member of the pBT2436-like megaplasmid family (>420 kb), normally carrying large arrays of antibiotic resistance genes in discrete, complex, and dynamic resistance regions. The members of this megaplasmid family normally share a common backbone, while plasmid pRBL16 lacks the AMR genes, implying it is quite flexible in the adaptive traits [31]. Furthermore, the concomitant distribution of *terCD* genes and the pathogenic related genes supported the relevance of tellurite resistance and microbial pathogenicity. It is noticed that strain SJTE-3ΔpRBL16 still showed tellurite resistance of about 40 μg/mL, which may be due to the existence of part of *arsABC* genes, *narGHI* genes and *cysK* gene in its genome [29,30,31]. Other genes for stress resistance factors in *Pseudomonas* spp. may also contribute to tellurite resistance of strain SJTE-3ΔpRBL16.

In addition, nanoparticles of elemental tellurium are useful in various fields like optic and electronic devices, and the Te nanoparticles reduced by microorganisms formed different structures such as nanorods, nanowires, nanotubes, and nanodots. *P. citronellolis* SJTE-3 has good environmental adaptability and robust performance, which can be used as a good candidate for tellurium bioproduction [25,26]. The nanostructures of tellurium formed in strain SJTE-3 cells are mostly uniform, spherical, or oval-shaped, with a diameter of about 100 nm, while the tellurium particles formed in *E. coli* cells were relatively loose and small. This may be due to higher tellurite concentration or stronger reductive enzyme activities in SJTE-3 cells. As most of the biosynthesized nanostructures of tellurium are wirelike, tubelike, and crystalline, the unusual vascular shape of tellurium nanostructures formed by strain SJTE-3 provided a new choice for the biosynthesis of this nanoparticle [2,55].

## 5. Conclusions

*P. citronellolis* SJTE-3 can tolerate tellurite of high concentrations, and *terZABCDE* gene cluster in plasmid pRBL16 was proved responsible for the high-level tellurite resistance of this strain. The *terCD* genes are necessary for high tellurite resistance with a great burden on cell growth, and *terA* or *terZ* genes could recover normal cell growth. The core tellurite-resistance genes (*terCD*) and the pathogenicity factor genes were frequently co-distribution. This work can promote the mechanism study of tellurite resistance and microbial pathogenicity.

## Figures and Tables

**Table 1:** The general features of plasmid pRBL16.

Features	Values
Length (bp)	370,338
ORF number	512
Longest ORF (bp)	5766
Average ORF length (bp)	649.18
Coding Region (bp)	332,382
GC content (%)	56.57
% of genome	89.75

**Table 2:** Tellurite resistance of the recombinant *E. coli* MG1655 containing different gene combinations of *terZABCDE* gene cluster in *P. citronellolis* SJTE-3.

	Tellurite Concentration (μg/mL)	1.0	2.0	5.0	10.0	20.0	50.0	100
Recombinant Strains								
MG1655 (pBS-terZABCDE)		+	+	+	+	+	+	+
MG1655 (pBS-terZABCE)		+	W	W	W	W	W	W
MG1655 (pBS-terZBCDE)		+	+	+	+	+	+	+
MG1655 (pBS-terZABDE)		W	−	−	−	−	−	−
MG1655 (pBS-terZACDE)		+	+	+	+	+	+	+
MG1655 (pBS-terZABCD)		+	+	+	+	+	+	+
MG1655 (pBS-terABCDE)		+	+	+	+	+	+	+
MG1655 (pBS-terABCD)		+	+	+	+	+	+	+
MG1655 (pBS-terBCDE)		+	+	+	+	+	+	+
MG1655 (pBS-terABCE)		+	+	−	−	−	−	−
MG1655 (pBS-terZACE)		+	+	+	+	+	+	−
MG1655 (pBS-terZACD)		+	+	+	+	+	+	+
MG1655 (pBS-terZABD)		−	−	−	−	−	−	−
MG1655 (pBS-terZADE)		W	W	−	−	−	−	−
MG1655 (pBS-terZCDE)		+	+	+	+	+	+	+
MG1655 (pBS-terZABE)		−	−	−	−	−	−	−
MG1655 (pBS-terZBDE)		+	W	W	−	−	−	−
MG1655 (pBS-terABDE)		W	−	−	−	−	−	−
MG1655 (pBS-terACDE)		+	+	+	+	+	+	+
MG1655 (pBS-terZBCD)		+	+	+	+	+	+	+
MG1655 (pBS-terZBCE)		+	+	W	W	W	W	W
MG1655 (pBS-terZABC)		−	−	−	−	−	−	−
MG1655 (pBS-terZAB)		−	−	−	−	−	−	−
MG1655 (pBS-terZAC)		−	−	−	−	−	−	−
MG1655 (pBS-terZAD)		−	−	−	−	−	−	−
MG1655 (pBS-terZAE)		−	−	−	−	−	−	−
MG1655 (pBS-terZBC)		−	−	−	−	−	−	−
MG1655 (pBS-terZBD)		−	−	−	−	−	−	−
MG1655 (pBS-terZBE)		−	−	−	−	−	−	−
MG1655 (pBS-terZCD)		+	+	+	+	+	+	W
MG1655 (pBS-terZCE)		W	W	W	W	W	−	−
MG1655 (pBS-terZDE)		−	−	−	−	−	−	−
MG1655 (pBS-terABC)		−	−	−	−	−	−	−
MG1655 (pBS-terABD)		−	−	−	−	−	−	−
MG1655 (pBS-terABE)		−	−	−	−	−	−	−
MG1655 (pBS-terACD)		+	+	+	+	+	+	+
MG1655 (pBS-terACE)		+	+	+	+	W	−	−
MG1655 (pBS-terADE)		−	−	−	−	−	−	−
MG1655 (pBS-terBCD)		+	+	+	W	W	−	−
MG1655 (pBS-terBCE)		W	W	W	W	−	−	−
MG1655 (pBS-terBDE)		−	−	−	−	−	−	−
MG1655 (pBS-terCDE)		W	−	−	−	−	−	−
MG1655 (pBS-terZA)		−	−	−	−	−	−	−
MG1655 (pBS-terZB)		−	−	−	−	−	−	−
MG1655 (pBS-terZC)		−	−	−	−	−	−	−
MG1655 (pBS-terZD)		−	−	−	−	−	−	−
MG1655 (pBS-terZE)		−	−	−	−	−	−	−
MG1655 (pBS-terAB)		−	−	−	−	−	−	−
MG1655 (pBS-terAC)		−	−	−	−	−	−	−
MG1655 (pBS-terAD)		−	−	−	−	−	−	−
MG1655 (pBS-terAE)		−	−	−	−	−	−	−
MG1655 (pBS-terBC)		−	−	−	−	−	−	−
MG1655 (pBS-terBD)		−	−	−	−	−	−	−
MG1655 (pBS-terBE)		−	−	−	−	−	−	−
MG1655 (pBS-terCD)		W	W	−	−	−	−	−
MG1655 (pBS-terCE)		W	−	−	−	−	−	−
MG1655 (pBS-terDE)		−	−	−	−	−	−	−
MG1655 (pBSPPc)		−	−	−	−	−	−	−
MG1655		−	−	−	−	−	−	−

To determine the cell growth and tellurite resistance properties of the recombinant strains, the minimal inhibitory concentration (MIC) of tellurite to cells was determined using the agar and broth dilution methods, and the cell growth was assessed after incubation for a defined period of time [28,29]. The growth curves were monitored with an Automated Microbiology Growth Curve Analyzer (Bioscreen, Oy Growth Curves Ab Ltd., Helsinki, Finland) at 37 °C for 24 h. The recombinant strains cells and the wild-type MG1655 cells were also screened on the tellurite-amended plates. The strain able to grow on the solid plates with tellurite of certain concentration and the OD600 value reaching 0.2 in the liquid medium with tellurite after 24 h, was considered able to resist tellurite of specific concentration. Three independent experiments were performed and the average values were presented. +, positive growth; −, negative growth; W, small and slow-growing colonies.

## Data Availability

Not applicable.

## Supplementary Materials

The following are available online at https://www.mdpi.com/article/10.3390/microorganisms10010095/s1, Table S1: Strains and plasmids, Table S2: Oligonucleotides used in this study, Table S3: The MIC values of different bacteria to tellurite.

## References

[1] Belzile N., Chen Y. (2015). Tellurium in the environment: A critical review focused on natural waters, soils, sediments and airborne particles. Appl. Geochem..

[2] He Z., Yang Y., Liu J.-W., Yu S.-H. (2017). Emerging tellurium nanostructures: Controllable synthesis and their applications. Chem. Soc. Rev..

[3] Xie H.G., Xia W., Chen M., Wu L.C., Tong J. (2018). Isolation and Characterization of the tellurite-reducing photosynthetic bacterium, *Rhodopseudomonas palustris* strain TX618. Water Air Soil Pollut..

[4] Shen J., Jia S., Shi N., Ge Q., Gotoh T., Lv S., Zhu M. (2021). Elemental electrical switch enabling phase segregation-free operation. Science.

[5] Ba L.A., Döring M., Jamier V., Jacob C. (2010). Tellurium: An element with great biological potency and potential. Org. Biomol. Chem..

[6] Vaigankar D.C., Dubey S.K., Mujawar S.Y., D’Costa A., Shyama S.K. (2018). Tellurite biotransformation and detoxification by *Shewanella baltica* with simultaneous synthesis of tellurium nanorods exhibiting photo-catalytic and anti-biofilm activity. Ecotoxicol. Environ. Saf..

[7] Chasteen T.G., Fuentes D.E., Tantaleán J.C., Vásquez C.C. (2009). Tellurite: History, oxidative stress, and molecular mechanisms of resistance. FEMS Microbiol. Rev..

[8] Perez J.M., Calderón I.L., Arenas F.A., Fuentes D.E., Pradenas G.A., Fuentes E.L., Sandoval J.M., Castro M.E., Elías A.O., Vásquez C.C. (2007). Bacterial toxicity of potassium tellurite: Unveiling an ancient enigma. PLoS ONE.

[9] Calderón I.L., Arenas F.A., Pérez J.M., Fuentes D.E., Araya M.A., Saavedra C.P., Tantaleán J.C., Pichuantes S.E., Youderian P.A., Vásquez C.C. (2006). Catalases are NAD(P)H-dependent tellurite reductases. PLoS ONE.

[10] Borsetti F., Tremaroli V., Michelacci F., Borghese R., Winterstein C., Daldal F., Zannoni D. (2005). Tellurite effects on *Rhodobacter capsulatus* cell viability and superoxide dismutase activity under oxidative stress conditions. Res. Microbiol..

[11] Turner R.J., Weiner J.H., Taylor D.E. (1995). The tellurite-resistance determinants tehAtehB and klaAklaBtelB have different biochemical requirements. Microbiology.

[12] Fuentes D.E., Fuentes E.L., Castro M.E., Pérez J.M., Araya M.A., Chasteen T.G., Pichuantes S.E., Vásquez C.C. (2007). Cysteine metabolism-related genes and bacterial resistance to potassium tellurite. J. Bacteriol..

[13] Moore M.D., Kaplan S. (1992). Identification of intrinsic high-level resistance to rare-earth oxides and oxyanions in members of the class Proteobacteria: Characterization of tellurite, selenite, and rhodium sesquioxide reduction in *Rhodobacter sphaeroides*. J. Bacteriol..

[14] Avazéri C., Turner R.J., Pommier J., Weiner J.H., Giordano G., Verméglio A. (1997). Tellurite reductase activity of nitrate reductase is responsible for the basal resistance of *Escherichia coli* to tellurite. Microbiology.

[15] O’Gara J.P., Gomelsky M., Kaplan S. (1997). Identification and molecular genetic analysis of multiple loci contributing to high-level tellurite resistance in *Rhodobacter sphaeroides* 2.4.1. Appl. Environ. Microbiol..

[16] Cournoyer B., Watanabe S., Vivian A. (1998). A tellurite-resistance genetic determinant from phytopathogenic *pseudomonads* encodes a thiopurine methyltransferase: Evidence of a widelyconserved family of methyltransferases. Biochim. Biophys. Acta.

[17] Tantaleán J.C., Araya M.A., Saavedra C.P., Fuentes D.E., Pérez J.M., Calderón I.L., Youderian P., Vásquez C.C. (2003). The *Geobacillus stearothermophilus* V iscS gene, encoding cysteine desulfurase, confers resistance to potassium tellurite in *Escherichia coli* K-12. J. Bacteriol..

[18] Taylor D.E. (1999). Bacterial tellurite resistance. Trends Microbiol..

[19] Zeinert R., Martinez E., Schmitz J., Senn K., Usman B., Anantharaman V., Aravind L., Waters L.S. (2018). Structure-function analysis of manganese exporter proteins across bacteria. J. Biol. Chem..

[20] Dambach M., Sandoval M., Updegrove T.B., Anantharaman V., Aravind L., Waters L.S., Storz G. (2015). The ubiquitous yybP-ykoY riboswitch is a manganese-responsive regulatory element. Mol. Cell.

[21] Guzzo J., Dubow M.S. (2000). A novel selenite- and tellurite-inducible gene in *Escherichia coli*. Appl. Environ. Microbiol..

[22] Chiang S.K., Lou Y.C., Chen C. (2008). NMR solution structure of KP-TerB, a tellurite-resistance protein from *Klebsiella pneumoniae*. Protein Sci..

[23] Pan Y.-R., Lou Y.-C., Seven A.B., Rizo J., Chen C. (2011). NMR structure and calcium-binding properties of the tellurite resistance protein TerD from *Klebsiella pneumoniae*. J. Mol. Biol..

[24] Bhatia M., Girdhar A., Tiwari A., Nayarisseri A. (2014). Implications of a novel *Pseudomonas* species on low density polyethylene biodegradation: An in vitro to in silico approach. Springerplus.

[25] Zheng D., Wang X., Wang P., Peng W., Ji N., Liang R. (2016). Genome sequence of *Pseudomonas citronellolis* SJTE-3, an estrogen- and polycyclic aromatic hydrocarbon-degrading bacterium. Genome Announc..

[26] Peng W., Fu Y., Jia B., Sun X., Wang Y., Deng Z., Lin S., Liang R. (2022). Metabolism analysis of 17α-ethynylestradiol by *Pseudomonas citronellolis* SJTE-3 and identification of the functional genes. J. Hazard. Mater..

[27] Kumar S., Stecher G., Li M., Knyaz C., Tamura K. (2018). MEGA X: Molecular evolutionary genetics analysis across computing platforms. Mol. Biol. Evol..

[28] Davison H.C., Woolhouse M.E., Low J.C. (2000). What is antibiotic resistance and how can we measure it?. Trends Microbiol..

[29] Wiegand I., Hilpert K., Hancock R.E.W. (2008). Agar and broth dilution methods to determine the minimal inhibitory concentration (MIC) of antimicrobial substances. Nat. Protoc..

[30] Morales E.H., Pinto C.A., Luraschi R., Muñoz-Villagrán C.M., Cornejo F.A., Simpkins S.W., Nelson J., Arenas F.A., Piotrowski J.S., Myers C.L. (2017). Accumulation of heme biosynthetic intermediates contributes to the antibacterial action of the metalloid tellurite. Nat. Commun..

[31] Cazares A., Moore M.P., Hall J.P.J., Wright L.L., Grimes M., Emond-Rhéault J.-G., Pongchaikul P., Santanirand P., Levesque R.C., Fothergill J.L. (2020). A megaplasmid family driving dissemination of multidrug resistance in *Pseudomonas*. Nat. Commun..

[32] Turner R.J., Hou Y., Weiner J.H., Taylor D.E. (1992). The arsenical ATPase efflux pump mediates tellurite resistance. J. Bacteriol..

[33] Alonso G., Gomes C., González C., Rodríguez Lemoine V. (2000). On the mechanism of resistance to channel-forming colicins (PacB) and tellurite, encoded by plasmid Mip233 (IncHI3). FEMS Microbiol. Lett..

[34] Berks B.C., Richardson D.J., Robinson C., Reilly A., Aplin R.T., Ferguson S.J. (1994). Purification and characterization of the periplasmic nitrate reductase from *Thiosphaera pantotropha*. Eur. J. Biochem..

[35] Whelan K.F., Sherburne R.K., Taylor D.E. (1997). Characterization of a region of the IncHI2 plasmid R478 which protects *Escherichia coli* from toxic effects specified by components of the tellurite, phage, and colicin resistance cluster. J. Bacteriol..

[36] Taylor D.E., Rooker M., Keelan M., Ng L.K., Martin I., Perna N.T., Burland N.V., Blattner F.R. (2002). Genomic variability of O islands encoding tellurite resistance in enterohemorrhagic *Escherichia coli* O157:H7 isolates. J. Bacteriol..

[37] Nguyen T.T.H., Kikuchi T., Tokunaga T., Iyoda S., Iguchi A. (2021). Diversity of the tellurite resistance gene operon in *Escherichia coli*. Front. Microbiol..

[38] Whelan K.F., Colleran E., Taylor D.E. (1995). Phage inhibition, colicin resistance, and tellurite resistance are encoded by a single cluster of genes on the IncHI2 plasmid R478. J. Bacteriol..

[39] Liu M., Turner R.J., Winstone T.L., Saetre A., Dyllick-Brenzinger M., Jickling G., Tari L.W., Weiner J.H., Taylor D.E. (2000). *Escherichia coli* TehB requires S-adenosylmethionine as a cofactor to mediate tellurite resistance. J. Bacteriol..

[40] Borsetti F., Borghese R., Cappelletti M., Zannoni D. (2018). Tellurite processing by cells of *Rhodobacter capsulatus* involves a periplasmic step where the oxyanion causes a malfunction of the cytochrome C maturation system. Int. Biodeterior. Biodegrad..

[41] SVávrová S., Struhárňanská E., Turňa J., Stuchlík S. (2021). Tellurium: A rare element with influence on prokaryotic and eukaryotic biological systems. Int. J. Mol. Sci..

[42] Vornhagen J., Bassis C.M., Ramakrishnan S., Hein R., Mason S., Bergman Y., Sunshine N., Fan Y., Holmes C.L., Timp W. (2021). A plasmid locus associated with *Klebsiella* clinical infections encodes a microbiome-dependent gut fitness factor. PLoS Pathog..

[43] Vávrová S., Valkova D., Drahovská H., Kokavec J., Mravec J., Turna J. (2006). Analysis of the tellurite resistance determinant on the pNT3B derivative of the pTE53 plasmid from uropathogenic *Escherichia coli*. Biometals.

[44] Turkovicova L., Smidak R., Jung G., Turna J., Lubec G., Aradska J. (2016). Proteomic analysis of the TerC interactome: Novel links to tellurite resistance and pathogenicity. J. Proteom..

[45] Kwon K.C., Cho M.H. (2008). Deletion of the chloroplast-localized at TerC gene product in *Arabidopsis thaliana* leads to loss of the thylakoid membrane and to seedling lethality. Plant J..

[46] Schneider A., Steinberger I., Strissel H., Kunz H.H., Manavski N., Meurer J., Burkhard G., Jarzombski S., Schünemann D., Geimer S. (2014). The Arabidopsis Tellurite resistance C protein together with ALB3 is involved in photosystem II protein synthesis. Plant. J..

[47] Sanssouci E., Lerat S., Grondin G., Shareck F., Beaulieu C. (2011). tdd8: A TerD domain-encoding gene involved in *Streptomyces coelicolor* differentiation. Antonie Leeuwenhoek.

[48] Valkova D., Valkovičová L., Vávrová S., Kováčová E., Mravec J., Turna J. (2007). The contribution of tellurite resistance genes to the fitness of *Escherichia coli* uropathogenic strains. Open Life Sci..

[49] Ponnusamy D., Clinkenbeard K.D. (2015). Role of tellurite resistance operon in filamentous growth of *Yersinia pestis* in macrophages. PLoS ONE.

[50] Yin X., Wheatcroft R., Chambers J.R., Liu B., Zhu J., Gyles C.L. (2009). Contributions of O island 48 to adherence of enterohemorrhagic *Escherichia coli* O157:H7 to epithelial cells in vitro and in ligated pig ileal loops. Appl. Environ. Microbiol..

[51] Tarr P.I., Bilge S.S., Vary J.C., Jelacic S., Habeeb R.L., Ward T.R., Baylor M.R., Besser T.E. (2000). Iha: A novel *Escherichia coli* O157:H7 adherence-conferring molecule encoded on a recently acquired chromosomal island of conserved structure. Infect. Immun..

[52] Azeddoug H., Reysset G. (1994). Cloning and sequencing of a chromosomal fragment from *Clostridium acetobutylicum* strain ABKn8 conferring chemical-damaging agents and UV resistance to *E. coli* recA strains. Curr. Microbiol..

[53] Ponnusamy D., Hartson S.D., Clinkenbeard K.D. (2011). Intracellular *Yersinia pestis* expresses general stress response and tellurite resistance proteins in mouse macrophages. Vet. Microbiol..

[54] Poirier K., Faucher S., Béland M., Brousseau R., Gannon V., Martin C., Harel J., Daigle F. (2008). *Escherichia coli* O157:H7 survives within human macrophages: Global gene expression profile and involvement of the Shiga toxins. Infect. Immun..

[55] Wu S., Li T., Xia X., Zhou Z., Zheng S., Wang G. (2019). Reduction of tellurite in *Shinella* sp. WSJ-2 and adsorption removal of multiple dyes and metals by biogenic tellurium nanorods. Int. Biodeterior. Biodegrad..

