# Supplementary material for: Characterization of the Tellurite-Resistance Properties and Identification of the Core Function Genes for Tellurite Resistance in Pseudomonas citronellolis SJTE-3
Source: Microorganisms. 2022 Jan 1;10(1):95. doi: 10.3390/microorganisms10010095 (PMC8779313; doi:10.3390/microorganisms10010095)
Supplement: Supplementary file 1 [file microorganisms-10-00095-s001.zip › Table S1 Strains and plasmids used in this study-1222.pdf]

Table S1 Strains and plasmids

Name	Description	Source/ Reference
**Strains**		
P. citronellolis SJTE-3	Strain with tellurite resistance, wild type	[25]
P. citronellolis SJTE-3 Δ pRBL16	Strain SJTE-3 without plasmid pRBL16	This study
E. coli DH5α	F′/*endA1 hsdR*17 (rK$^-$mK$^-$) *glnV*44 *thi*$^-$ *rec*A1 *gyr*A (*nalR*) *rel*A1 Δ(*lacIZYA-argF*) U169 *deo*R (Φ80d*lac*Δ (*lacZ*)M15)	Invitrogen
E. coli MG1655	F$^-$ λ$^-$ *ilvG*$^-$ *rfb*$^-$ *rph*$^{-1}$	Invitrogen
**Plasmids**		
pBSPPC-Gm	*oriT*$^+$gene replacement vector derived from pBR322, Ap^r, Gm^r	[1]
pBS-Pter	Plasmid pBSPPC inserted with the promoter of *terZABCDE* gene cluster	This study
pBS-*terZABCDE*	Plasmid pBS-Pter inserted with *terZABCDE* gene cluster	This study
pBS-*terZABCE*	Plasmid pBS-Pter inserted with *terZABCE* genes	This study
pBS-*terZBCDE*	Plasmid pBS-Pter inserted with *terZBCDE* genes	This study
pBS-*terZABDE*	Plasmid pBS-Pter inserted with *terZABDE* genes	This study

pBS-*terZACDE*	Plasmid pBS-Pter inserted with *terZACDE* genes	This study
pBS-*terZABCD*	Plasmid pBS-Pter inserted with *terZABCD* genes	This study
pBS-*terABCDE*	Plasmid pBS-Pter inserted with *terABCDE* genes	This study
pBS-*terABCD*	Plasmid pBS-Pter inserted with *terABCD* genes	This study
pBS-*terBCDE*	Plasmid pBS-Pter inserted with *terBCDE* genes	This study
pBS-*terABCE*	Plasmid pBS-Pter inserted with *terABCE* genes	This study
pBS-*terZACE*	Plasmid pBS-Pter inserted with *terZACE* genes	This study
pBS-*terZACD*	Plasmid pBS-Pter inserted with *terZACD* genes	This study
pBS-*terZABD*	Plasmid pBS-Pter inserted with *terZABD* genes	This study
pBS-*terZADE*	Plasmid pBS-Pter inserted with *terZADE* genes	This study
pBS-*terZCDE*	Plasmid pBS-Pter inserted with *terZCDE* genes	This study
pBS-*terZABE*	Plasmid pBS-Pter inserted with *terZABE* genes	This study
pBS-*terZBDE*	Plasmid pBS-Pter inserted with *terZBDE* genes	This study
pBS-*terABDE*	Plasmid pBS-Pter inserted with *terABDE* genes	This study
pBS-*terACDE*	Plasmid pBS-Pter inserted with *terACDE* genes	This study
pBS-*terZBCD*	Plasmid pBS-Pter inserted with *terZBCD* genes	This study
pBS-*terZBCE*	Plasmid pBS-Pter inserted with *terZBCE* genes	This study
pBS-*terZABC*	Plasmid pBS-Pter inserted with *terZABC* genes	This study
pBS-*terZAB*	Plasmid pBS-Pter inserted with *terZAB* genes	This study
pBS-*terZAC*	Plasmid pBS-Pter inserted with *terZAC* genes	This study
pBS-*terZAD*	Plasmid pBS-Pter inserted with *terZAD* genes	This study

pBS-*terZAE*	Plasmid pBS-Pter inserted with *terZAE* genes	This study
pBS-*terZBC*	Plasmid pBS-Pter inserted with *terZBC* genes	This study
pBS-*terZBD*	Plasmid pBS-Pter inserted with *terZBD* genes	This study
pBS-*terZBE*	Plasmid pBS-Pter inserted with *terZBE* genes	This study
pBS-*terZCD*	Plasmid pBS-Pter inserted with *terZCD* genes	This study
pBS-*terZCE*	Plasmid pBS-Pter inserted with *terZCE* genes	This study
pBS-*terZDE*	Plasmid pBS-Pter inserted with *terZDE* genes	This study
pBS-*terABC*	Plasmid pBS-Pter inserted with *terABC* genes	This study
pBS-*terABD*	Plasmid pBS-Pter inserted with *terABD* genes	This study
pBS-*terABE*	Plasmid pBS-Pter inserted with *terABE* genes	This study
pBS-*terACD*	Plasmid pBS-Pter inserted with *terACD* genes	This study
pBS-*terACE*	Plasmid pBS-Pter inserted with *terACE* genes	This study
pBS-*terADE*	Plasmid pBS-Pter inserted with *terADE* genes	This study
pBS-*terBCD*	Plasmid pBS-Pter inserted with *terBCD* genes	This study
pBS-*terBCE*	Plasmid pBS-Pter inserted with *terBCE* genes	This study
pBS-*terBDE*	Plasmid pBS-Pter inserted with *terBDE* genes	This study
pBS-*terCDE*	Plasmid pBS-Pter inserted with *terCDE* genes	This study
pBS-*terZA*	Plasmid pBS-Pter inserted with *terZA* genes	This study
pBS-*terZB*	Plasmid pBS-Pter inserted with *terZB* genes	This study
pBS-*terZC*	Plasmid pBS-Pter inserted with *terZC* genes	This study
pBS-*terZD*	Plasmid pBS-Pter inserted with *terZD* genes	This study
pBS-*terZE*	Plasmid pBS-Pter inserted with *terZE* genes	This study

pBS-*terAB*	Plasmid pBS-Pter inserted with *terAB* genes	This study
pBS-*terAC*	Plasmid pBS-Pter inserted with *terAC* genes	This study
pBS-*terAD*	Plasmid pBS-Pter inserted with *terAD* genes	This study
pBS-*terAE*	Plasmid pBS-Pter inserted with *terAE* genes	This study
pBS-*terBC*	Plasmid pBS-Pter inserted with *terBC* genes	This study
pBS-*terBD*	Plasmid pBS-Pter inserted with *terBD* genes	This study
pBS-*terBE*	Plasmid pBS-Pter inserted with *terBE* genes	This study
pBS-*terCD*	Plasmid pBS-Pter inserted with *terCD* genes	This study
pBS-*terCE*	Plasmid pBS-Pter inserted with *terCE* genes	This study
pBS-*terDE*	Plasmid pBS-Pter inserted with *terDE* genes	This study

[1] Xu, Y., Tao, F., Ma, C., and Xu, P. (2013). New constitutive vectors: useful genetic engineering tools for biocatalysis. *Appl Environ Microbiol* 79(8), 2836-2840. doi: 10.1128/AEM.03746-12.
