# Supplementary material for: Characterization of the Tellurite-Resistance Properties and Identification of the Core Function Genes for Tellurite Resistance in Pseudomonas citronellolis SJTE-3
Source: Microorganisms. 2022 Jan 1;10(1):95. doi: 10.3390/microorganisms10010095 (PMC8779313; doi:10.3390/microorganisms10010095)
Supplement: Supplementary file 1 [file microorganisms-10-00095-s001.zip › Table S2. Oligonucleotides used in this study-1222.pdf]

Table S2. Oligonucleotides used in this study

Name	Sequences (5’-3’)	Usage
terZ-F/R	GGGtctagaGATCAACAAGGAGCGTAAACATGGCACTCACC, CCAGTGTTGTCATGCGCGTA	Primers used for *terZ* amplification
terA-F/R	GGGtctagaCCTCGTCAACCTGGCCATTCA, GGGggatccTTACTTGGAGCCCGCTTTCCAG	Primers used for *terA* amplification
terB-F/R	GGGtctagaAAAGCGGGCTCCAAGTAAAACTTAT, GGGggatccCTTACAGTTCGAAGTCTGCCGG	Primers used for *terB* amplification
terC-F/R	GGGtctagaCGAACTGTAAGGAACTGAAACGTGG, GGGggatccATTACTGGTTTTCGGCTTGCGC	Primers used for *terC* amplification
terD-F/R	GGGtctagaTCAGTAGCAACCACAAGGAGCTG, GGGggatccTGAGGGATCAGTTGACGGTCAGACCGTAACC	Primers used for *terD* amplification
terE-F/R	TCCCTCACCCCAACTGAATCAAG, GGGggatccCTTAGCCGATACTCACGCCGTGAGC	Primers used for *terE* amplification
terZB	ATAAGTTTTACTTGGAGCCCGCTTTCCAGTGTTGTCATGCGCGTA	Primer used for *terZ* and *terB* overlap
terZC	CCACGTTTCAGTTCCTTACAGTTCGCCAGTGTTGTCATGCGCGTA	Primer used for *terZ* and *terC* overlap
terZD	TACAGCTCCTTGTGGTTGCTACTGACCAGTGTTGTCATGCGCGTA	Primer used for *terZ* and *terD* overlap
terZE	TCCTTGATTCAGTTGGGGTGAGGGACCAGTGT	Primer used for *terZ*

	TGTCATGCGCGTA	and *terE* overlap
terAC	CCACGTTTCAGTTCCTTACAGTTCGTTACTTGG AGCCCGCTTTCCAG	Primer used for *terA* and *terC* overlap
terAD	TACAGCTCCTTGTGGTTGCTACTGATTACTTGG AGCCCGCTTTCCAG	Primer used for *terA* and *terD* overlap
terAE	TCCTTGATTCAGTTGGGGTGAGGGATTACTTGG AGCCCGCTTTCCAG	Primer used for *terA* and *terE* overlap
terBD	TACAGCTCCTTGTGGTTGCTACTGACTTACAGT TCGAAGTCTGCCGG	Primer used for *terB* and *terD* overlap
terBE	TCCTTGATTCAGTTGGGGTGAGGGACTTACAG TTCGAAGTCTGCCGG	Primer used for *terB* and *terE* overlap
terCE	TCCTTGATTCAGTTGGGGTGAGGGAATTACTG GTTTTCGGCTTGCGC	Primer used for *terC* and *terE* overlap

The uppercase letters represented the sequences for amplification and the lowercase letters represented the sequences for restriction enzyme digestion; the underlined letters were the protecting bases.
