# Supplementary material for: Characterization of the Tellurite-Resistance Properties and Identification of the Core Function Genes for Tellurite Resistance in Pseudomonas citronellolis SJTE-3
Source: Microorganisms. 2022 Jan 1;10(1):95. doi: 10.3390/microorganisms10010095 (PMC8779313; doi:10.3390/microorganisms10010095)
Supplement: Supplementary file 1 [file microorganisms-10-00095-s001.zip › Table S3 MIC of different species to tellurite-1222.pdf]

Table S3. The MIC values of different bacteria to tellurite

Strains	Plasmid	MIC (μg/ml)	References
Escherichia coli JE2571	None	3	[1]
Escherichia coli JE2571	RP4	4	[1]
Escherichia coli JE2571	$RP4Te^r$	325	[1]
Escherichia coli JE2571	pIN25	4	[1]
Escherichia coli JE2571	F	4	[1]
Escherichia coli JE2571	R478	240	[1]
Escherichia coli JE2571	MIP233	100	[1]
Escherichia coli JE2571	pMG110	175	[1]
Escherichia coli JE2571	pMUR162	175	[1]
Serratia marcescens ATCC 13880	None	10	[1]
Serratia marcescens ATCC 13880	$RP4Te^r$	250	[1]
Pseudomonas aeruginosa PA01 150.1	None	75	[1]
Pseudomonas aeruginosa PA01 150.1	RP4	75	[1]
Pseudomonas aeruginosa PA01 150.1	$RP4Te^r$	300	[1]
Pseudomonas aeruginosa PA01 150.1	CAM	100	[1]
Pseudomonas aeruginosa PA01 150.1	OCT	100	[1]
Pseudomonas aeruginosa PA01 150.1	RPL11	90	[1]
Pseudomonas putida PpS388	None	6	[1]
Pseudomonas putida PpS388	$RP4Te^r$	375	[1]
Alcaligenes odorans Clinical	None	30	[1]

Alcaligenes odorans Clinical	RP4Te^r	475	[1]
Acinetobacter calcoaceticus C91	None	175	[1]
Acinetobacter calcoaceticus C91	RP4Te^r	300	[1]
Klebsiella aerogenes	pHH1508a	512	[2]
Pseudomonas aeruginosa	RK2	256	[3]
Escherichia coli HB101	R773	64	[29]

[1] D.E. Bradley, K.K. Grewal, et al. Characteristics of RP4 tellurite-resistance transposon Tn521. *J Gen Microbiol* **1988**, *134*, 2009-2018. doi: 10.1099/00221287-134-7-2009

[2] D.E. Bradley, V.M. Hughes, et al. R plasmids of a new incompatibility group determine constitutive production of H pili. *Plasmid* **1982**, *7*, 230-238. doi: 10.1016/0147-619x(82)90004-x.

[3] E.G. Walter, C.M. Thomas, et al. Transcriptional analysis, translational analysis, and sequence of the kilA-tellurite resistance region of plasmid RK2Ter. *J Bacteriol* **1991**, *173*, 1111-1119. doi: 10.1128/jb.173.3.1111-1119.1991.
